# Chloride-Induced Corrosion of Steel in Alkali-Activated Mortars Based on Different Precursors

**DOI:** 10.3390/ma13225244

**Published:** 2020-11-20

**Authors:** Antonino Runci, Marijana Serdar

**Affiliations:** Department of Materials, Faculty of Civil Engineering, University of Zagreb, 10000 Zagreb, Croatia; antonino.runci@grad.unizg.hr

**Keywords:** alkali-activated mortar, corrosion, chloride, pore structure, chloride migration

## Abstract

The low environmental impact and high long-term performance of products are becoming imperative for the sustainable development of the construction industry. Alkali-activated materials (AAMs) are one of the available low-embodied-carbon alternatives to Portland cement (OPC). For their application in the marine environment or where de-icing salts are used, it is of utmost importance to demonstrate their equal or better performance compared to OPC. The aim of this study was to compare the corrosion behaviour of the steel in AAMs based on different regionally available by-products with the behaviour of the steel in OPC. The by-products used were fly ash, slag, silica fume, and iron-silica fines. The corrosion process of each system was monitored by the corrosion potential and polarisation resistance during exposure to tap water and chloride solution over a period of almost one year. Certain AAMs showed a higher resistance to chloride penetration compared to OPC, which was attributed to the smaller number of capillary pores and higher gel phase precipitation. The same corrosion resistance compared to OPC was achieved with alkali-activated fly ash and alkali-activated slag mortars. The stability of the systems in tap water and chloride solution was confirmed by the visual assessment of the steel surface at the end of the test period.

## 1. Introduction

The growing need for more sustainable materials and the urgent need to manage the landfilling of the industrial waste materials found in alkali-activated materials (AAMs) require a valid simultaneous answer. AAMs are a group of alternative clinker-free binders that have been developed over the last century. The renewed interest in AAMs is motivated by their valorisation of by-products from other industrial activities and their lower CO_2_ emissions compared to Ordinary Portland Cement (OPC) [1]. The reduction in CO_2_ emissions with AAMs is achieved by avoiding the clinkerisation process needed in OPC production. OPC production currently accounts for 8% of the global anthropological CO_2_ emissions, with a total annual production of 3.6 billion tons. The growing demand may increase the production to 3.7–5.5 Gt per year [2]. The production process of AAMs might lead to savings in greenhouse gas emission compared to OPC of around 44–64% [3]. The main emissions in the case of AAMs come from activator production and the transportation and pre-treatment of precursors. According to Habert et al. [4], despite certain challenges related to other outstanding environmental impact categories and by-product supplies, the utilisation of AAMs could be one of the available technologies providing a sharp CO_2_ reduction.

Alkali-activated materials (AAMs) consist of aluminosilicate powder, usually by-products such as blast furnace slag from steel production or fly ash from a coal-fired plant, and an alkali-activator based on Na or K, commonly used as hydroxide or silicate liquid solution. The alkali activation is a chemical process which includes the dissolution/reprecipitation reactions of amorphous aluminosilicate powders, resulting in the formation of solid products similar to the hydration products in cement paste or ceramics [1]. AAMs can generally be divided into two different groups based on the composition of the reaction phases [5]: (i) low Ca systems based on the activation of a low Ca precursor, such as fly ash or metakaolin, with the main reaction product being a three-dimensional gel of the alkali-aluminosilicate hydrate (N-A-S-H) type; and (ii) high Ca systems based on the activation of a high Ca precursor, such as blast furnace slag, with the main reaction product being a gel of the calcium-aluminosilicate-hydrate (C-A-S-H) type.

AAMs have already shown many engineering characteristics comparable to those of OPC, and published studies report a satisfying compressive strength [6], a higher stability when exposed to elevated temperature [7], a higher resistance against chemical attack [8], and potentially better resistance to freeze-thaw cycles [9] compared to OPC. Ensuring the durability of AAMs in the marine environment or where de-icing salts are used is one of the main milestones for their wider practical application. In fact, the main cause of the early degradation of reinforced concrete is chloride ingress and the resulting steel corrosion [10]. Previous research on AAMs has indicated the possibility of applying AAMs in the corrosive environment. Babaee and Castel [11] have tested the main possible combinations of fly ash and blast furnace slag with different solution moduli (Ms = molar ratio SiO_2_/Na_2_O) of silicate-based activators and NaOH concentrations. Their research showed that increasing the alkali concentration in the activator and using sodium silicate activator with an Ms of 1.5 gave a desirable microstructure which improved the physical barrier to chloride penetration [12]. Research performed on AAMs with high sulphide concentration, such as systems based on blast furnace slag, showed a reduction in the redox potential at steel concrete interface area [13], without signs of steel depassivation. Still, such a reduction in potential drastically influences the final electrochemical parameters [14] and makes the evaluation of the corrosion behaviour of steel in AAMs using the limiting values established for OPC challenging.

As relatively new materials, the quantity of available durability data on AAMs is limited, and the long-term performance of structures made with AAMs is yet to be determined. Available knowledge of the degradation mechanisms of traditional reinforced concrete cannot be directly transferred to AAMs due to the difference in reaction products, pore solution chemistry, and microstructure of the matrix. Additionally, what makes any generalisation on the durability of AAMs even more challenging is the wide chemical variability of precursors and high impact of the type and quantity of alkali activator used.

In this study, different alkali-activated mortars were developed using regionally available materials as precursors. The aim of the study was to evaluate the influence of differently developed alkali-activated mortars on the corrosion behaviour of steel in a simulated marine environment. The corrosion behaviour was monitored using linear polarisation measurement. The ability of mortars to resist chloride penetration was analysed and correlated to their pore structure. Finally, the influence of mortar composition on corrosion risk was correlated to its ability to provide physical protection to the steel.

## 2. Materials and Methods

### 2.1. Raw Materials

Four different regionally available materials were used as precursors to develop alkali-activated mortars: (i) a commercial ground granulated blast-furnace slag (BFS) from the iron production process supplied by Lafarge Holcim (Koromačno, Croatia); (ii) fly ash (FA) from a coal-fired power plant by Elektroprivreda (Kakanj, Bosnia and Herzegovina); (iii) silica fume (SF) from silicon metal production by R-D Silicon D.O.O. (Zenica, Bosnia and Herzegovina); and (iv) iron-silicate fine (IS) from copper production by Aurubis (Pirdop, Bulgaria). Mortar based on Ordinary Portland Cement (OPC) CEM I supplied by CEMEX (Kaštel Sućurac, Croatia) was used as a reference material. Table 1 shows the chemical composition of the raw materials and Portland cement used, obtained by X-ray fluorescence (XRF).

While slag, fly ash, and silica fume are relatively common precursors, in the present study an alternative precursor from copper production was also used, in the form of iron silica fines. Iron silica fines have a different mineralogical composition than other used by-products; it mostly contains fayalite, magnetite, and Fe-diopside. Another particularity of iron silica fine is its particle size distribution. Similar to silica fume, iron silica fines have very fine particles, with the median D (v,0.5) diameter of 0.4 µm compared to the average of 10 µm for the cement and fly ash used. The precursors were activated with sodium silicate Geosil 34,417 from Woellner (Ludwigshafen am Rhein, Germany) with an Ms = 1.68 and NaOH 17.8 M solution or potassium silicate Geosil 14,515 from Woellner (Ludwigshafen am Rhein, Germany) with an Ms = 1.5. The aggregate was local dolomite sand with a granulometry of 0–4 mm. The binder/aggregate ratio used was 0.3.

### 2.2. Mix Designs

Table 2 summarises the mix design of developed AAMs. Mortars labelled S and FA are pure systems based only on blast furnace slag and fly ash, respectively, inspired by the International Union of Laboratories and Experts in Construction Materials, Systems and Structures RILEM TC 247-DTA [15]. The mortar labelled S_FA is a mix of 75% blast furnace slag and 25% fly ash based on the publication by Babaae and Castel [11]. The mortar labelled IS_S is a system based on iron-silicate fines with blast furnace slag, and it is the only mix activated by potassium silicate. This mix was based on the work by Provis et al. [16], where iron-silicate fines in combination with slag have a shown good performance as a precursor of alkali-activated concrete. Since iron silicate fines are available in large quantities, the substitution of slag in the alkali-activated systems could yield economic and ecologic benefits. Finally, the mortar labelled S_SF is a system based on blast furnace slag with 15% silica fume based on [17].

AAMs were prepared according to the RILEM TC 247-DTA [15] recommendation: Dry powders and aggregate were mixed for a minute, and then again for 6 min while continuously adding activators; the mixing was paused for a minute and then the mixture was mixed faster for an additional minute. Samples were demoulded after one day and were then tightly wrapped to prevent moisture loss for continued sealed curing until testing. The reference mortar (labelled REF) was based on CEM I with a water/binder ratio of 0.4 and a binder/aggregate ratio of 0.3, prepared according to EN 196-1. Samples of reference mortar were cured in the humidity chamber until testing.

### 2.3. Methods

The compressive strength of the mortar specimens after 28 days of curing was determined on prisms 4 × 4 × 16 cm according to EN 196-1 with a loading rate of 2400 kN. The non-steady-state chloride migration was conducted according to NT BUILD 492 [18]. Three specimens per mix with a 100 mm diameter and 50 mm height were tested after 28 days of curing. AgNO_3_ colorimetric analysis was applied at the end of the test. The chloride penetration depth was measured from the white silver chloride precipitation (AgCl) when chloride is present in sufficient quantities (otherwise there is the precipitation of brown Ag_2_O), after which the chloride migration coefficient can be calculated using an integrated form of the Nerst–Planck equation:

(1) $$D_{nssm}=\frac{0.0239\cdot \left(273+T\cdot L\right)}{\left(U-2\right)\cdot t}\cdot \left(x_{d}-0.0238\cdot \sqrt{\frac{\left(237+T\right)\cdot L\cdot x_{d}}{U-2}}\right),$$

where *D_nssm_* is the chloride migration coefficient in ×10^−12^ m^2^/s, *U* is the applied voltage in V, *T* is the average of the initial and final temperature in °C, *L* is the thickness of the sample in mm, *x_d_* is the average penetration depth in mm, and *t* is the test duration in h. An important parameter for this test is the chloride concentration at which the colour changes (*c_d_*). The NT BUILD 492 recommends the value of 0.07 N for *c*_d_ for the OPC concrete. However, for AAMs *c_d_* = 0.21 was applied [19,20] to take into account hydroxide ions (pH value). In fact, silver nitrate can react with both chloride ions and hydroxyl ions to form a white silver chloride (AgCl) and a dark brown silver oxide (Ag_2_O). When aqueous silver nitrate solution is sprayed on the surface of the split concrete, the precipitates formed on the surface of concrete are a mixture of AgCl and Ag_2_O. For each chemistry of the pore solution, there is a critical point where the brown colour of the silver oxide covers the white colour of the silver chloride and forms the penetration front. The concentration of chloride ions at this critical point (penetration front) is taken as *c*_d_. Indeed, the silver nitrate colorimetric measurement is based on a chemical reaction, which might be influenced by the chemistry of the concrete pore solution. The fact that alkali-activated materials have a different chemistry of pore solution, which is not considered in the NT BUILD 492 method, is accommodated by a difference in the *c*_d_ parameter.

Mercury Intrusion porosimetry (MIP) was used to provide information regarding the pore size distribution and pore volume of the mortars [21]. The specimens for MIP were immersed in isopropanol for 14 days and then vacuum dried. Autopore IV 9510 was used for the MIP measurement, with a maximum pressure of 208 MPa and a pore size ranging from 350 to 0.007 µm. The pore diameter was derived using Washburn’s law:

(2) $$D=\frac{(-4\cos\theta )\cdot \gamma }{P},$$

where *D* is the pore diameter (μm), *θ* is the contact angle between the fluid and the pore mouth (130°), *γ* is the surface tension of the fluid (485 mN/m), and *P is* the applied pressure to fill up the pore with mercury (MPa).

To detect the alkaline condition of mortar, the pH was measured through the leaching of alkali metals. Every 7 days, 5 g of mortar fine powder was diluted in 50 g of distilled water. The pH of the leaching solution was measured at 24 h after the powder addition. The pH of the obtained leachate solution was deemed to be an acceptable approximation of the pH of the mortar pore electrolyte [22]. Parallel to pH measurement, the electrical resistivity of the mortar was measured using the Wenner probe.

**Figure 1:**
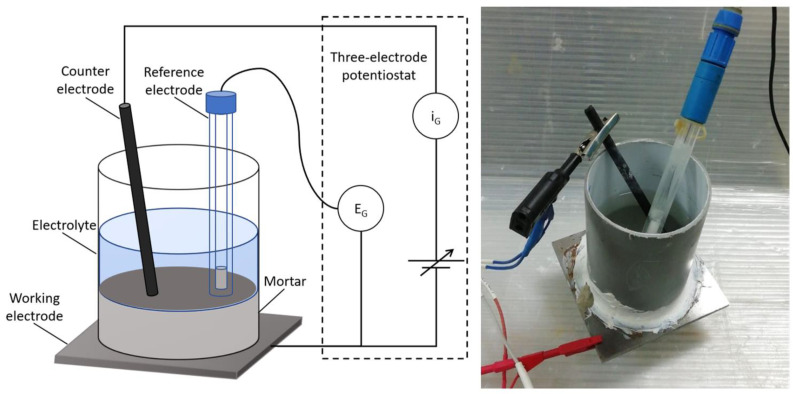
Schematic representation of the three-electrode cell.

The monitoring of the corrosion of steel in mortar was carried out using an unconventional three-electrode cell suggested by Šoić et al. [23] with a PAR VMP2 potentiostat/galvanostat (Biologic, Seyssinet-Pariset, France). The samples were prepared using a carbon steel plate as working electrodes, which simulated the reinforcement inside the concrete. Carbon steel plates were polished until an even metallic surface was obtained. On top of the steel plate, a polymeric cylinder was glued with silicon. This cylinder was used as a mould for the prepared mortar. A graphite stick was placed inside the solution to act as the counter electrode, and a saturated calomel electrode (SCE, SI Analytics, Mainz, Germany) was positioned to act as the reference electrode (Figure 1).

The samples were cured for 7 days, after which tap water (reference exposure) or 3.5% NaCl water solution (chloride solution exposure) were added to each cell (3 samples per each exposure and per each mix). Samples were covered with plastic foil to prevent electrolyte evaporation in the period between measurements. The open-circuit potential (OCP) and linear polarisation resistance (LPR) were measured, respectively, for 15 and 1 min. For the LPR measurements, the steels were polarised to ±10 mV of corrosion potential (*E_corr_*) at a scan rate of 0.166 mV/s. The polarisation resistance (*R_p_*) was calculated by the slope of the polarisation curve using the modified Stern–Geary equation:

(3) $$R_{p}={\left(\frac{\Delta E}{\Delta I}\right)}_{\Delta E\to 0},$$

(4) $$i_{corr}=\frac{B}{R_{p}}$$

In a potentiodynamic test, Δ*E* is the potential step applied within a limited range of overpotentials (*η* = *E* − *E_corr_* = −10 to +10 mV in the current study) to polarise the corrosion system, Δ*I* is the system response to the potential excitation, *i_corr_* is the corrosion current density (A/cm^2^), and B is the proportionality constant (V) depending on the Tafel constant. Corrosion current densities were calculated from the measured *R_p_* values, using the theoretical value of Tafel constant of B = 52 mV. This value of Tafel constant is usually used for passive steel in the OPC system. However, in a lack of available measurements of Tafel constants in AAMs systems, the same value was taken hereafter. The *R_p_* is considered as the sum of resistances of the electrolyte solution, mortar, and polarised steel without *IR* compensation, as is described by Equation (5):

(5) $$E=E_{eq}+\eta +IR,$$

where *E_eq_* is the equilibrium potential defined by the Nernst equation, *η* is the overvoltage related to the exchanged current measured with respect to the equilibrium potential, and *IR* is ohmic drop in the electrolyte solution and mortar. Here, it was considered that the electrical resistivity of a testing solution and all mortars is equal for each set of samples (exposed to water and exposed to 3.5 wt.% NaCl), which is surely an oversimplification.

## 3. Results

### 3.1. Mortar Characterisation

Table 3 shows the mortar slump, compressive strength, and porosity of the alkali-activated mortars cured in sealed conditions for 28 days and the reference OPC mortar cured in a humidity chamber.

**Figure 2:**
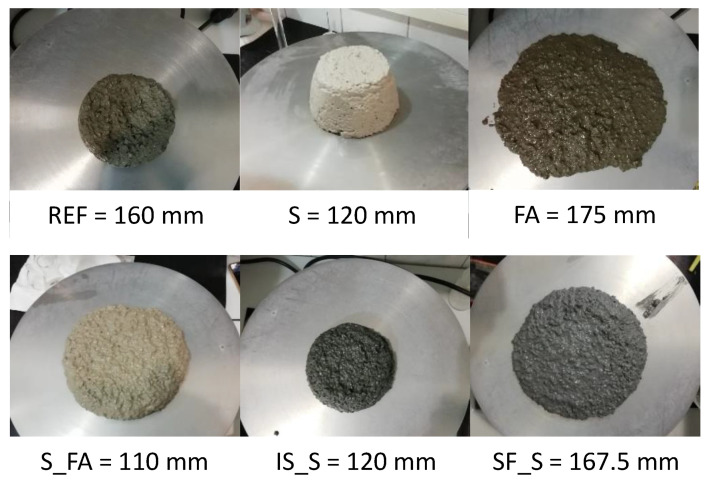
Visual observation of the slump for different mortars.

Most of the AAMs displayed lower slump values than the referent mortar due to the lower water/binder ratio and higher reactivity of the systems (Figure 2). FA and S_SF mixes were the only two AAM mortars showing higher slump results. However, the higher slump of mortar based on fly ash (mortar FA) was somewhat misleading, since the mix was relatively difficult to compact. The systems containing fly ash showed also the highest compressive strength, compared to the other mixes, due to the high activator content and the lower water/binder ratio.

**Figure 3:**
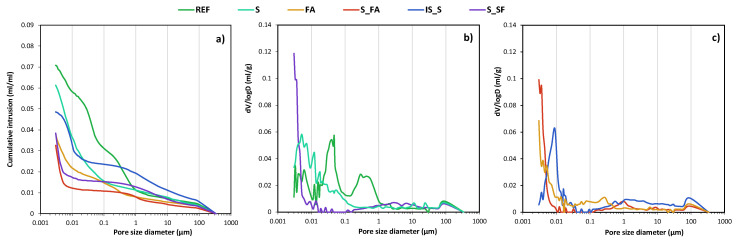
(**a**) Pore size distribution cumulative curve and (**b**,**c**) pore size distribution differential curve at 28 days.

The pore size distributions of the mortars were assessed by MIP analysis after 28 days of curing, and the results are presented in Figure 3. Three different pore size regions can be distinguished: gel pores below 0.015 μm (which do not affect permeability); capillary pores ranging between 0.015 and 0.5 μm (affecting fluid permeability and mechanical properties [24]); and macropores with radii ≥ 0.5 μm, mainly the influencing mechanical properties [25]. The figure shows that the main difference between the reference mix and the AAMs was the capillary pore range. While the mortar based on OPC showed a progressive increase and visible peaks between 1 and 0.2 μm and around 0.05 μm, the AAMs showed a low amount of macropores and capillary pores and a sharp increase in the range of gel pores with pores smaller than 0.01 μm. The only exception is the IS_S system, which showed a heterogeneous pores size distribution with a significant porosity between macropores and capillary pores.

**Figure 4:**
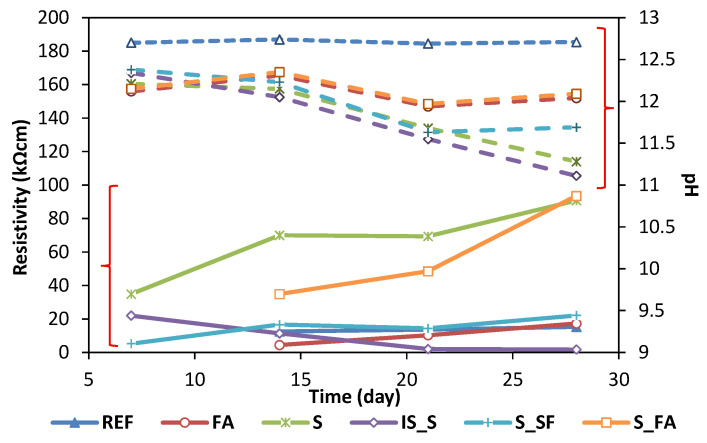
Evolution of the surface resistivity (continue line) and pH of the leaching solution (dotted line).

The pH is the main factor influencing the stability of a passive film on a steel surface. Figure 4 shows the pH values of mixes at 7, 14, 21, and 28 days of curing compared with resistivity values, measured at the same age of the mortar. The reference mix has the highest pH value of around 12.7, compared to all alkali-activated mortars. Instead, the pH value of the AAMs started at 12.1–12.3 and maintained a stable value of 12.1 in the system containing fly ash, while the value dropped to 11.7 and 11.2 for S_SF mortar and both S and IS_S mortar, respectively. All the mixes showed an increase in resistivity up to 14 days, except for IS_S mortar, for which the value was slightly decreasing over time. Mortars S and S_FA showed the highest values of resistivity, up to 90 kΩcm, compared to around 20 kΩcm for all other systems. A high increase in the resistivity of mix S was followed by a significant pH drop due to the dense structure and lower activator content. Mixes S_FA and FA, while having similar trends in terms of pH values, which were decreasing over time, had different behaviour in terms of resistivity values. For mix S_FA, the resistivity was significantly increasing, while for mix FA the increase in resistivity during aging was not found to be significant.

**Figure 5:**
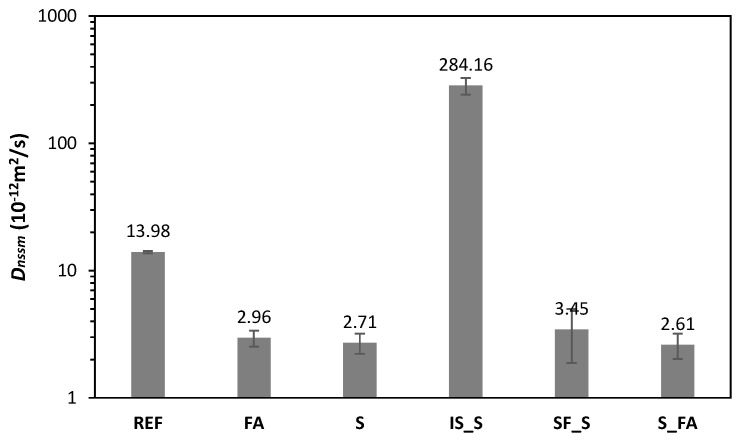
Apparent chloride migration coefficient measured after 28 days curing for all mixes according to NT BUILD 492.

Figure 5 shows the apparent chloride migration coefficients of all mortars, measured and calculated according to NT BUILD 492. Comparing the values of the chloride migration coefficients, all the alkali-activated mortars had lower chloride migration coefficients compared to the reference mortar. IS_S represents the only exception, showing an extremely high chloride migration coefficient. Mortar S_FA showed the best performance. While comparing to the RILEM data, the apparent chloride diffusion of S is coherent with the RILEM round-robin test observation, while the coefficient of the FA mix is significantly lower than expected for the alkali-activated fly ash mix.

### 3.2. Corrosion Monitoring

**Figure 6:**
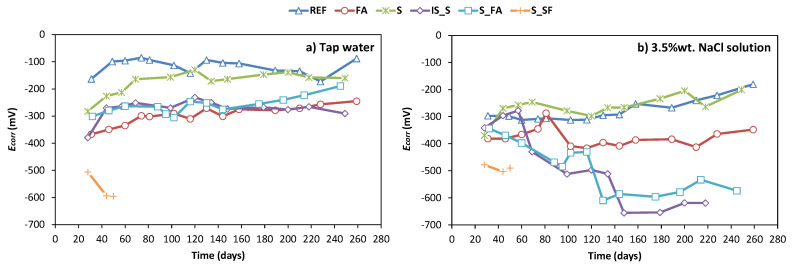
Open-circuit potential of steel embedded in mortar immersed in (**a**) tap water and (**b**) NaCl 3.5 wt.% solution, with 7 days of curing prior to immersion or exposure.

Figure 6a shows the results of the open-circuit potential (*E_corr_*) of steel embedded in mortar exposed to tap water as testing solution and Figure 6b shows those exposed to 3.5% NaCl water solution as testing solution. All the results shown in these figures were obtained as an average value of three identical samples.

In the case of reference sample under tap water, the reference mortar showed a standard behaviour; mainly, after a small increase in *E_corr_* from −160 till −100 mV at 45 days, the potential stabilised between −150 and −100 mV during the entire testing period (250 days of testing). In a corrosive environment, the *E_corr_* was expectedly lower compared to exposure in tap water and remained stable at −300 mV up to 250 days of testing. The initial values of potential in the case of mix S were lower compared to the reference mix; values started from −300 mV in tap water and −460 mV in chloride solution. Nevertheless, the values were gradually increasing and stabilised at around −200 mV under tap water and −300 mV under 3.5% NaCl water solution, which was similar to the behaviour observed with the reference mix. In the case of mixes FA, S_FA, and IS_S, the *E_corr_* in tap water was slightly more negative, compared to the reference and S mix, but still stayed stable around −300 mV throughout the testing period. The steel in mix FA exposed to chloride solution also stayed stable at −400 mV throughout the testing period, even though the value of potential was slightly lower compared to the potential in reference mix and compared to FA mix in tap water. On the other hand, the potentials of mixes S_FA and IS_S during exposure to chloride solution were constantly decreasing and reached values of −650 mV at the end of the testing period. Finally, mix S_SF showed unexpected behaviour in both tap water and exposure to chloride solution. The *E_corr_* values of steel in this mix were constantly very low, below −500 mV. What was realised during testing was that, for mix S_SF, the mortar detached from the mould, which led to a crevice formation on the side of the sample. Furthermore, the surface of the mortar was covered with cracks, which were potentially also allowing the penetration of testing solution and allowing its direct contact with the surface of the steel. It was therefore the case that the monitoring of the corrosion behaviour for this mix was abandoned after 60 days of testing.

**Figure 7:**
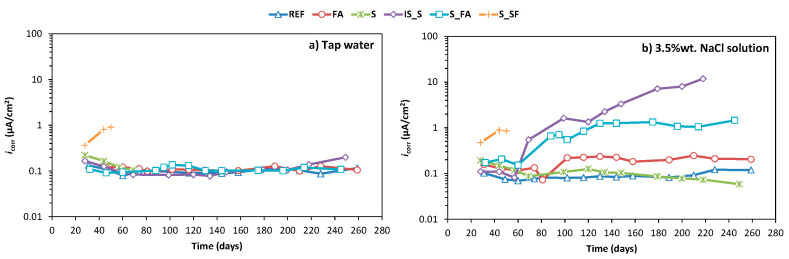
Current density of steel embedded in mortar immersed in (**a**) tap water and (**b**) NaCl 3.5 wt.% solution, with 7 days of curing prior to immersion or exposure.

Figure 7a shows the results of the current density (*i_corr_*) of steel embedded in mortar exposed to tap water as testing solution, and Figure 7b shows those exposed to 3.5% NaCl water solution as a testing solution. All the results shown in these figures were obtained as an average value of three identical samples.

Looking at the values of current density over time in the reference cell (tap water exposure), it can be observed that the values remained stable between 0.07 and 0.13 µA/cm^2^ for all the tested mixes. Here, also, mix SF_S was the only system showing *i_corr_* values higher than 0.5 µA/cm^2^. In the corrosive environment (exposure to chloride solution), the current density values of reference mortar (REF), FA and S were stable between 0.07 and 0.25 µA/cm^2^ over time, which is in accordance with stable corrosion potential. Mixes IS_S and S_FA had a similar behaviour until 60 days, after which the *i_corr_* was increasing in time constantly until it reached a value of 11.8 µA/cm^2^ for IS_S and 1.5 µA/cm^2^ for S_FA. Similar to the value in tap water, the SF_S mix showed a high value of current density immediately after the exposure.

**Figure 8:**
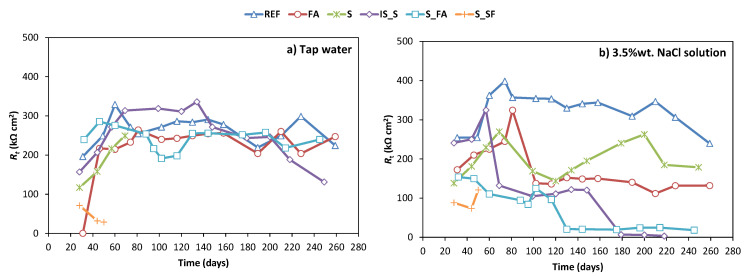
Total resistance of steel embedded in mortar immersed in (**a**) tap water and (**b**) NaCl 3.5 wt.% solution, with 7 days of curing prior to immersion or exposure.

Figure 8a shows the results of the total resistivity (*R_t_*) of steel embedded in mortar exposed to tap water as testing solution and Figure 8b shows those exposed to 3.5% NaCl water solution as testing solution. All the results shown in these figures were obtained as an average value of three identical samples.

In the reference cell (tap water exposure), the resistivity value of all systems, except for SF_S, increased from the time of measurement until 60–80 days of curing, where it stabilised between 200 and 350 kΩ·cm^2^. For SF_S mix, the resistivity was decreasing immediately after exposure from 70 to 30 kΩ·cm^2^. After 200 days, resistivity of IS_S mix started to decrease to 120 kΩ·cm^2^. This decrease in resistivity was in accordance with a slight increase in current density and decrease in potential, evident for this mix at the same testing time.

In the corrosive cell (chloride solution exposure), the resistivity of reference mortar remained stable at a value of around 350 kΩ·cm^2^. For the mixes S and FA, the values of resistivity were increasing during initial exposure, but then stabilised at slightly lower values of 200 kΩ·cm^2^. The resistivity values for mixes S_FA and IS_S were continuously decreasing during exposure and reached value of less than 50 kΩ·cm^2^ at the end of the testing period. Finally, the SF_S values were from the beginning of exposure very low, which is in accordance with the potential and current density values and was explained earlier.

### 3.3. Steel Surface Analyses

**Figure 9:**
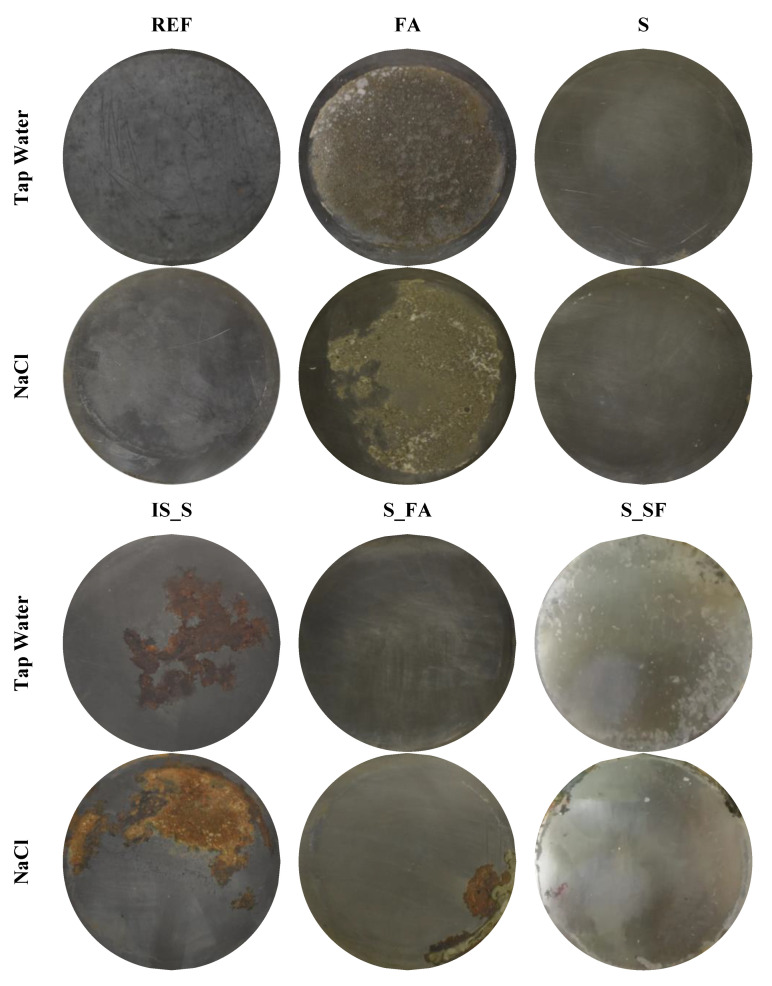
Visual appearance of steel surface at the end of testing, exposed to tap water and chloride solution.

After the electrochemical measurement, the steel plates were separated from the mortar and visually assessed for corrosion. The steel surface was shallowly cleaned with HCl and ethanol before assessment. Figure 9 shows the appearance of steel surface after exposure in mortar to tap water (upper image) and NaCl 3.5 wt.% solution (lower image). In REF, FA and S systems, no rust layers were visible neither in tap nor in NaCl environment. The FA mix was strongly adherent to the steel surface, despite the smoothness of the steel, and it was not possible to remove the remaining mortar layer. In the S_FA mix, the rust layer is visible only under NaCl exposure closer to the contact between the mortar and the polyethylene mould. This corrosion attack could be attributed to crevice corrosion. The steel in the mix IS_S showed a wide corrosion layer in both environments at the end of the testing period. Finally, the steel in the SF_S mix had corrosion products all around the contact between the mortar and the polyethylene mould already after 50 days of curing under tap water and NaCl environment, which confirmed the hypothesis that the extremely high values of current density and low values of potential were attributed to a direct contact between the steel and the testing solution.

## 4. Discussion

The electrochemical stability of steel embedded in concrete can be affected by many aspects. In Portland cement, it depends mostly on the water/binder ratio, which influences the physical ability of cement matrix to resist SO_4_^2−^, Cl^−^, CO_2_, and H^+^ penetration and chloride binding capacity, on pH of pore solution and on the steel surface conditions [26]. In AAMs, the type of precursor, the type and quantity of the activator used, and the curing conditions represent the main aspects influencing the concrete microstructure, as well as the pH and composition of the pore solution. These parameters are prevailing for evaluating the ability of the matrix to provide a stable environment for the formation of a passive film on the surface of the steel and to provide a physical barrier to chloride penetration. Therefore, these parameters will hereafter be correlated to the corrosion behaviour of steel in developed and tested AAM systems.

In this research, the chloride diffusion coefficient according to NT BUILD 492 demonstrated the higher resistance of AAMs to chloride ingress compared to the reference mix, except for the mix based on slag and iron-silica fines (IS_S). Alkali-activated slag mix, developed based on a design from RILEM TC 247 [27], showed values similar to those obtained by laboratories participating in this technical committee. For low-Ca systems based on fly ash, generally around two orders of magnitude-higher values of chloride migration than those of high-Ca systems are expected. Contrary to what was expected, the alkali-activated fly ash system presented in this paper showed lower chloride migration values than the same system tested in RILEM TC 247 [27]. Furthermore, the blended fly ash slag system outperformed the slag system when observing the chloride migration values. The low values of chloride migration for mix with fly ash can be explained by a high Ca content of used fly ash, which was almost double of that from RILEM TC 247 [15]. A high Ca content is fundamental for the precipitation of C-A-S-H gel which is the main reaction product in high-Ca alkali-activated systems, such as systems based on blast furnace slag. C-A-S-H has high density structure that offers an efficient barrier to chloride diffusion. C-A-S-H gel systems have shown lower porosity and higher tortuosity, reducing the diffusion coefficient [28]. Reducing the calcium content, the chloride diffusion increases because the C-A-S-H gel is substituted by N-A-S-H gel, the main reaction product of low-Ca systems. On the other hand, N-A-S-H gel favours more than C-A-S-H the adsorption of chloride ions [29], especially if it has a low Ca/Si ratio due to the higher surface area and the differences in aluminosilicate framework charge [30]. The precipitation of halite (NaCl) has been identified in dried paste specimens [11,20], which got adsorbed or encapsulated within the geopolymer matrix [19]. It is therefore possible that in the blended fly ash-slag system developed here a simultaneous effect of pore filler of C-A-S-H gel and binding capacity on N-A-S-H gel surface [31] was obtained, which led to a lower chloride migration coefficient in the blended system compared to both pure fly ash and pure slag systems. In their previous research, Ganeshan and Venkataraman [32] also observed lower water permeability and chloride diffusion in systems based on fly ash class C. The structure of the system looked denser and with less voids because of the precipitation of mixed C-(N-)A-S-H gel.

Substituting 15% of blast furnace slag with silica fume did not show the expected positive effect on chloride migration, opposite to what was demonstrated by Behfarnia and Rostami [17]. A total of 15% of silica fume, however, did result in extreme compressive strength and reduced sharply the capillary pores, potentially due to the C-A-S-H gel filling the empty space between the particles in the mortar matrix [17,33]. Even though the chloride migration of this mix was still lower than that of the OPC mix, extremely high corrosion parameters were obtained. It seems that such a high addition of very fine silica fume particles led to extensive shrinkage and consequently surface cracking, which resulted in the detachment of mortar from the mould. All of this finally led to the extremely poor corrosion performance of this system, which is potentially not realistic and will be dealt with more closely in the continuation of this research.

The system based on iron-silicate fine and blast furnace slag showed the lowest compressive strength after 28 days of curing. A low mortar resistivity indicates a possible low evolution of the matrix reaction, which directly influences the chloride resistance. Therefore, the lower reactivity of the iron-silicate fines justifies the higher chloride diffusion and lower gel pores compared to other AAMs [34]. However, steel in mortar based on iron-silicate fines and slag showed a stable passive behaviour during exposure in tap water, confirming the possible application of this system for reinforced concrete in a non-aggressive environment. For application in marine environment, a further optimisation of the mix design would be necessary.

The results of chloride migration positively correlate to the results of porosity of mortar at 28 days: system based on fly ash and blended fly ash-slag have lower porosity compared to system based on slag. In particular, the pore size distribution in fly ash and blended system is more focused on gel pores, while system based on slag has a gradual increase in pores from capillary to gel pores. Chloride migration results correlate also to the results of electrical resistivity of mortar. The resistivity of mixes based on slag and blended fly ash-slag system exhibited the highest values of electrical resistivity, confirming the higher pore refinement of C-A-S-H gel [19]. However, there is no correlation between the compressive strength and resistivity at 28 days for AAMs, differently from what was demonstrated for OPC [35] and for AAMs [19]. The chloride migration also shows a positive correlation to the total resistivity obtained from linear polarisation measurement, except for SF_F (for reasons explained earlier) and S_FA mixes. While S_FA showed extremely low values of chloride migration, it did exhibit significant corrosion activity during the testing period. The visual examination of the steel surface may explain this behaviour as a light detachment of mortar from the polypropylene mould and the formation of crevice corrosion, similar to the mix SF_S. This mechanical stability may be caused by the high liquid activator content for slag-based alkali-activated mortar [36,37]. However, this lower performance should also be investigated and confirmed with further research.

**Figure 10:**
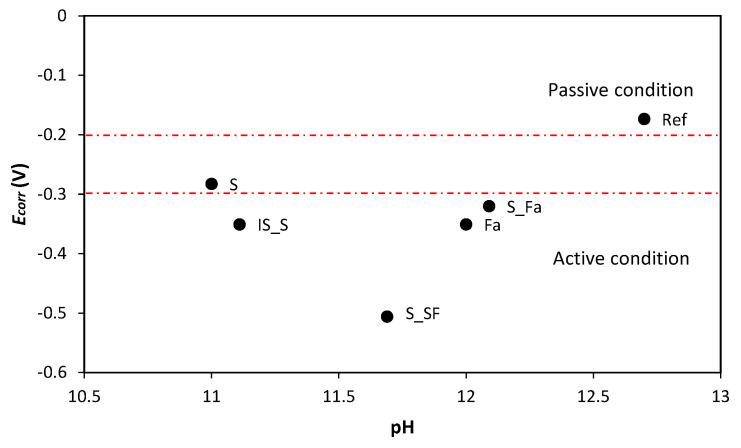
Correlation of the corrosion potential (V) of structural steel and pH of the leaching solution at 28 days of curing. The upper red line indicates > 90% probability of having no corrosion, while the bottom line indicates > 90% probability of having active corrosion, according to the standard ASTM C876 for steel in the OPC system [41].

The pH is the main parameter directly influencing the stability of passive film covering the steel surface. The initial high pH values of AAMs have demonstrated their ability to provide a protective passive film layer on the steel surface [38]. However, the cation reduction during the reaction [19,39], consumed during the geopolymerisation process, and the different chemical composition of pore solution may influence the stability of this passive layer. Noushimi et al. [19] have already demonstrated the higher stability of the pH values of OPC during curing compared to those of AAMs. Together with the lower pH values, the corrosion potentials of steel in AAMs under tap water were also lower compared to the OPC system. The potentials for steels in AAMs exposed to tap water were stabilised between −200 to −400 mV, while passive steel in OPC system showed a potential around −150 mV. This trend was already detected by Babaee et al. [40]. Figure 10 shows the correlations between the *E_corr_* and pH of AAMs and reference mortar at 28 days of curing.

AAMs show lower pH values and lower corrosion potential, and, even more, *E_corr_* and pH progressively reduce with increasing fly ash content. Several explanations are possible. First, cations could be leaching from fly ash-based systems, consequently reducing the pH of the alkali-activated binder [39,40]. Secondly, due to the low porosity of AAMs systems, leading to a low oxygen diffusion, there is potentially a low oxygen renewal possibility at the steel–concrete interface, which is fundamental to allow passive film formation. This aspect could be even further pronounced by the sulphide in blast furnace slag, which consumes the oxygen in the pore solution to be oxidated in sulphate. In fact, it was demonstrated that, in dry blast furnace slag, sulphur is mostly present as S^2−^ and S^0^ and then reacts with the oxygen and alkaline during the activation, forming S_2_O_3_ and SO_4_^2−^ [42,43]. The presence of sulphide species can significantly reduce the redox potential of the pore solution of slag-rich cement mortars [44], making the redox potential of slag-containing cements around 400 mV lower than that of OPC. Additionally, the presence of sulphide in the alkali-activated pore solution can directly influence the passive film and its stability, causing a less stable structure due to the competitive adsorption between HS^−^/S^2−^ and OH^−^ on the steel surface, disabling the formation of the passive film [38,45,46]. All this can be used to explain lower potential and also the slower formation and stabilisation of the passive film observed in the case of AAMs. While the reference mortar reached the passive conditions after 28 days, in AAMs the open-circuit potential was stabilised only after 40–50 days [11].

Together with the open-circuit potential, the total resistivity (*R_t_*) showed values outside the expected for OPC, according to the standard [41]. Traditionally, samples with *R_t_* values higher than 250 kΩ·cm^2^ are considered passive. The results presented here show that all AAM systems have a lower resistivity than 200 kΩ·cm^2^ in simulated sea water, while the OPC had *R_t_* values larger than 250 kΩ·cm^2^. Despite these lower values of resistivity and lower corrosion potentials, the current densities of alkali-activated mixes S and FA were as low as those of OPC. Furthermore, the visual examination of steel plates after the testing period confirmed that in these systems steel was passive in both tap water and in chloride solution, regardless of the potentials and resistivity being below the limiting values for OPC. These results highlight the challenges of transferring the same limiting values of corrosion parameters established for OPC to alkali-activated systems, since the same values for both systems will not necessarily indicate the same risk of corrosion activity.

## 5. Conclusions

The various characterisation techniques used in this work allow the following conclusions:

− The high compressive strength of an alkali-activated mortar cannot be the assurance of its ability to protect steel from corrosion in aggressive environments, which underlines the importance of conducting corrosion studies to evaluate the validity of a system.
− In general, alkali-activated mortars showed a lower chloride diffusion coefficient compared to OPC due to the higher mortar electrical resistivity and lower capillary porosity.
− The high degree of gel porosity indicates the increased degree of reaction of the mortar matrix, which leads to C-A-S-H and N-A-S-H gel precipitation, which could possibly be a reason for the lower chloride diffusion and higher chloride binding capacity.
− For certain mixes developed in this study, further optimisation of the mix design is required, focusing on the alkali activator and blast furnace slag content and assuring the volume stability of the matrix.
− The high electrochemical stability of most developed systems under tap water during the test period demonstrates the ability of AAMs to provide a stable passive environment for steel in a non-aggressive environment.
− The equal corrosion performance and better chloride penetration resistance of alkali-activated fly ash and alkali-activated slag mixture, compared to OPC, shows the applicability of these systems for concrete structures exposed to aggressive chloride-rich environments.

## Figures and Tables

**Table 1:** Chemical composition of the used materials.

	BFS	FA	SF	IS	OPC
CaO	33.46	13.04	3.06	2.7	64.04
SiO_2_	41.59	51.1	92.02	27.5	19.32
Al_2_O_3_	12.84	20.58	1.68	3.5	4.86
Fe_2_O_3_	0.73	7.42	0.45	66.5	2.94
Na_2_O	1.39	0.89	0.21	0.4	0.23
K_2_O	0.57	1.99	1.11	0.9	0.82
MgO	5.97	2.15	0.77	0.7	1.83
TiO_2_	1.73	0.53	0.04	0	0
MnO	0.08	0.04	0.03	0	0
SO_3_	1.65	1.72	0.27	0.7	2.75
P_2_O_5_	0.01	0.54	0.36	0	0

**Table 2:** Mix design of the mortars developed in this study.

Mix Label	Blast Furnace Slag	Fly Ash	Iron-Silicate Fine	Silica Fume	NaOH	Waterglass		Water
						KSi	NaSi	
S	100	0	0	0	8	0	6.2	33.6
FA	0	100	0	0	14	0	36.9	0
S_FA	75	25	0	0	9.5	0	13.8	8.8
IS_S	20	0	80	0	0	20	0	24
S_SF	85	0	0	15	15	0	24	21

**Table 3:** Mortar characteristics.

Mortar Property	REF	S	FA	S_FA	IS_S	S_SF
Slump (mm)	160	120	175	110	122.5	167.5
Compressive strength (MPa)	59.5	44.3	77.4	77.2	29.4	94.7
Total porosity (%)	21.0	18.4	11.5	9.6	14.3	8.7
